# Conditional Overexpression of Net1 Enhances the Trans‐Differentiation of Lgr5^+^ Progenitors into Hair Cells in the Neonatal Mouse Cochlea

**DOI:** 10.1111/cpr.13787

**Published:** 2024-12-15

**Authors:** Yanqin Lin, Qiuyue Zhang, Wei Tong, Yintao Wang, Leilei Wu, Hairong Xiao, Xujun Tang, Mingchen Dai, Zixuan Ye, Renjie Chai, Shasha Zhang

**Affiliations:** ^1^ State Key Laboratory of Digital Medical Engineering, Department of Otolaryngology Head and Neck Surgery, Zhongda Hospital, School of Life Sciences and Technology, Advanced Institute for Life and Health, Jiangsu Province High‐Tech Key Laboratory for Bio‐Medical Research Southeast University Nanjing China; ^2^ Southeast University Shenzhen Research Institute Shenzhen China; ^3^ Co‐Innovation Center of Neuroregeneration Nantong University Nantong China; ^4^ Institute for Stem Cell and Regeneration Chinese Academy of Science Beijing China; ^5^ Department of Otolaryngology Head and Neck Surgery, Sichuan Provincial People's Hospital University of Electronic Science and Technology of China Chengdu China

**Keywords:** HC regeneration, Lgr5^+^ progenitors, *Net1*, proliferation, trans‐differentiation

## Abstract

Sensorineural hearing loss is mainly caused by damage to hair cells (HC), which cannot be regenerated spontaneously in adult mammals once damaged. Cochlear Lgr5^+^ progenitors are characterised by HC regeneration capacity in neonatal mice, and we previously screened several new genes that might induce HC regeneration from Lgr5^+^ progenitors. *Net1*, a guanine nucleotide exchange factor, is one of the screened new genes and is particularly active in cancer cells and is involved in cell proliferation and differentiation. Here, to explore in vivo roles of Net1 in HC regeneration, *Net1* ^*loxp/loxp*^ mice were constructed and crossed with *Lgr5* ^*CreER/+*^ mice to conditionally overexpress (cOE) *Net1* in cochlear Lgr5^+^ progenitors. We observed a large number of ectopic HCs in *Lgr5* ^*CreER/+*^ *Net1* ^*loxp/loxp*^ mouse cochlea, which showed a dose‐dependent effect. Moreover, the EdU assay was unable to detect any EdU^+^/Sox2^+^ supporting cells, while lineage tracing showed significantly more regenerated tdTomato^+^ HCs in *Lgr5* ^*CreER/+*^ *Net1* ^*loxp/loxp*^ *tdTomato* mice, which indicated that *Net1* cOE enhanced HC regeneration by inducing the direct trans‐differentiation of Lgr5^+^ progenitors rather than mitotic HC regeneration. Additionally, qPCR results showed that the transcription factors related to HC regeneration, including *Atoh1*, *Gfi1* and *Pou4f3*, were significantly upregulated and are probably the mechanism behind the HC regeneration induced by *Net1*. In conclusion, our study provides new evidence for the role of *Net1* in enhancing HC regeneration in the neonatal mouse cochlea.

## Graphical abstract

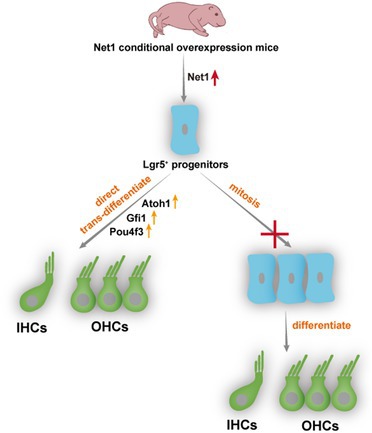

This work investigated the *in vivo* role of Net1 in cochlear hair cell (HC) regeneration. Conditional overexpression of Net1 in Lgr5^+^ progenitors of neonatal mouse cochlea enhance HC regeneration by inducing direct trans‐differentiation of Lgr5^+^ progenitors into HCs probably via activation of *Atoh1*, *Gfi1*, and *Pou4f3* transcription factors.

## 1 Introduction

The mammalian auditory sensory epithelium contains sensory hair cells (HCs) and non‐sensory supporting cells (SCs) that mediate the perception of sound. HCs consist of inner HCs (IHCs) and outer HCs (OHCs), while SCs are much more diverse, including inner border cells (IBCs), inner phalangeal cells (IPhCs), inner pillar cells (IPCs), outer pillar cells (OPCs), Deiters' cells (DCs) and Hensen's cells (HECs) [1, 2, 3]. HCs are susceptible to a variety of external factors, including ototoxic drugs, noise exposure and ageing [4, 5, 6], which can lead to HC damage and subsequent hearing loss. However, HCs cannot be spontaneously regenerated after damage to supplement HC loss in adult mammals [7, 8], which results in permanent hearing loss or deafness.

Fortunately, many studies have shown that SCs in newborn mammals have the ability to regenerate into HCs [7, 9, 10, 11]. Lgr5^+^ SCs, including the third‐row DCs, IPCs, IPhCs/IBCs and some lateral greater epithelial ridge (GER) cells, are considered to be progenitor cells with the capacity for HC regeneration in newborn mouse cochleae and in in vitro culture [12, 13, 14, 15, 16]. At present, it has been reported that several important genes and signalling pathways, such as the Wnt pathway [12, 13, 17], Notch pathway [18, 19, 20], Sonic hedgehog pathway [21], FGF signalling pathway [22], TGFβ pathway [23], *Atoh1* [24, 25, 26], *Foxg1* [27, 28], p27^Kip1^ [29, 30, 31], pRb [32, 33], *Rps14* [34, 35] and Bmi1 [36] are involved in HC regeneration from Lgr5^+^ progenitors in the neonatal mouse cochlea. Moreover, co‐regulation of two or more genes and signalling pathways has also been shown to further induce neonatal HC regeneration. In the mammalian cochlea, Notch inhibition induces mitosis in Lgr5^+^ progenitors and their differentiation into HCs through stimulation of the canonical Wnt pathway [19, 37]. The Wnt pathway and *Atoh1* [38] are co‐activated to promote Lgr5^+^ cell proliferation and differentiation in neonatal mice [25]. Besides, co‐activation of *Gfi1* [39], *Pou4f3* [40] and *Atoh1* (*GPA*) in cochlear SCs can significantly enhance HC regeneration and maturation [41]. Activation of *β‐catenin*, deletion of *Notch1* and overexpression of *Atoh1* in Sox2 SCs in vivo induces a large number of ectopic HCs in the neonatal mouse cochlea by up‐regulating the Wnt pathway and down‐regulating the Notch pathway [37, 42]. However, these regenerated HCs are still immature and have limited functional capacity [43, 44], and HCs still cannot be regenerated in adult mice [45]. Considering that HC regeneration may be a comprehensive process that involves cross‐talk between multiple genes and signalling pathways, it is of great significance to study novel genes that regulate HC regeneration and these genes' regulatory networks in order to further effect functional HC regeneration in neonatal and even adult mammals.

We previously screened several new genes that may have the ability to regulate HC regeneration by conducting joint analysis of RNA‐seq data sets [46, 47, 48] and in vitro sphere‐forming and differentiation assays (unpublished data). One of these genes is neuroepithelial transforming gene 1 (*Net1*), which encodes a guanine‐nucleotide exchange factor that can activate Rho‐GTPase, thus promoting the release of GDP and binding to GTP [49, 50]. RhoA/RhoB of the G‐protein superfamily is specifically activated by Net1 to regulate cell proliferation, differentiation and motility in response to various extracellular signals [51, 52]. Many studies have demonstrated that Net1 is highly enriched in a variety of human cancers and that it facilitates cell proliferation, differentiation and invasion by regulating actin cytoskeleton rearrangement and mitosis [53, 54, 55]. Knocking out *Net1* in breast cancer cells reduces tumour cell proliferation and promotes cell apoptosis [56], while up‐regulation of NET1 reverses the inhibition of baicalin on the proliferative and invasive capacity of lung cancer cells and enhances acute lymphoblastic leukaemia cell proliferation [57, 58]. Lysophosphatidic acid induction significantly increases Net1‐promoted gastric cancer cell migration and invasion [59]. In addition, Net1 has also been reported to regulate zebrafish mesoderm and embryonic development through the TGF‐β and canonical Wnt/β‐catenin signalling pathways [60, 61]. The above studies indicate that *Net1* mainly plays a regulatory role in cell proliferation, differentiation and development, but the function of *Net1* in the cochlea has not been described.

In this study, we conditionally overexpressed (cOE) *Net1* in Lgr5^+^ progenitors by constructing *Net1*‐floxp knock‐in mice and crossing them with *Lgr5* ^*CreER/+*^ mice in order to study the roles and mechanisms of Net1 on HC regeneration. We evaluated the proliferation and differentiation capacity of Lgr5^+^ progenitors and found that ectopic HCs increased after *Net1* cOE, and these were presumably trans‐differentiated directly from Lgr5^+^ progenitors. Moreover, our findings suggest that *Net1* cOE may promote trans‐differentiation of Lgr5^+^ progenitors into HCs via activation of *Atoh1*, *Gfi1* and *Pou4f3*, among which *Atoh1* may play a central role. Our findings have identified a novel gene involved in HC regeneration, and this will further promote research on rescuing hearing loss through functional HC regeneration.

## 2 Materials and Methods

### 2.1 Animals

**FIGURE 1:**
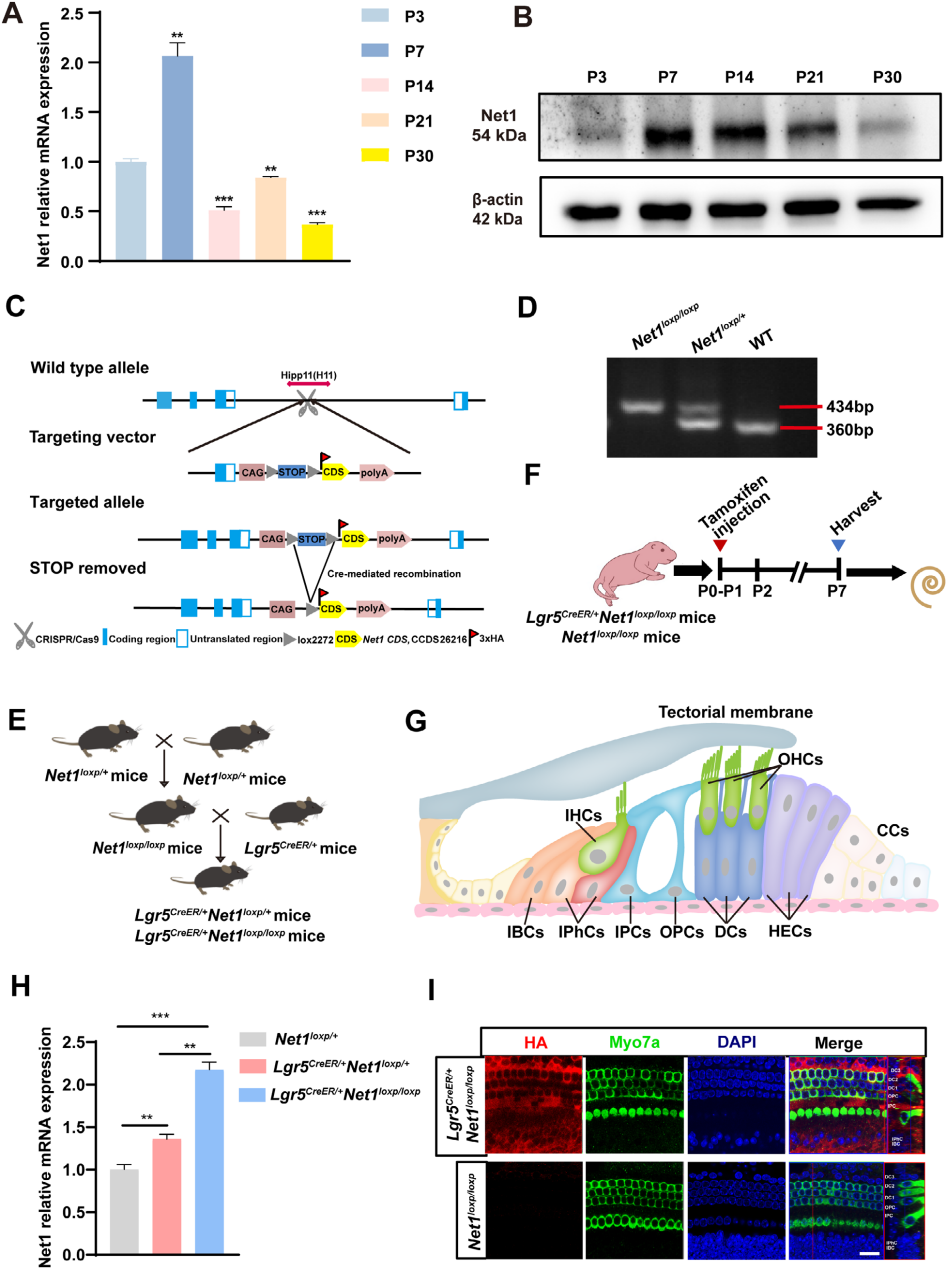
Conditional overexpression of *Net1* in Lgr5^+^ progenitors. (A, B) *Net1* mRNA and protein expression from P3 to P30 in the mouse cochlea were detected by real‐time qPCR (A) and Western blotting (B). (C) Schematic of the construction of C57BL/6J‐Hipp11‐loxp‐STOP‐loxp‐Net1 (*Net1* ^*loxp/+*^) transgenic mice. The *Net1* gene was expressed as a fusion with a 3× HA tag. (D, E) Breeding (E) and genotyping (D) of *Lgr5* ^*CreER/+*^ *Net1* ^*loxp/loxp*^ mice. (F) P0‐P1 mice were I.P. injected with tamoxifen (0.075 mg/g body weight) and the cochleae were harvested at P7 to determine the efficiency of *Net1* overexpression. (G) Schematic of cell types in the cochlea. OHCs, outer hair cells; IHCs, inner hair cells; IBCs, inner border cells; IPhCs, inner phalangeal cells; IPCs, inner pillar cells; OPCs, outer pillar cells; DCs, Deiters' cells; HECs, Hensen's cells; CCs, Claudius cells. (H, I) The *Net1* overexpression in *Net1* ^*loxp/+*^ mice, *Lgr5* ^*CreER/+*^ *Net1* ^*loxp/+*^ mice and *Lgr5* ^*CreER/+*^ *Net1* ^*loxp/loxp*^ mice was verified by real‐time qPCR (H). The 3× HA tag was stained (red) to identify overexpressed Net1 proteins in Lgr5^+^ progenitors of *Lgr5* ^*CreER/+*^ *Net1* ^*loxp/loxp*^ mice, and SCs types are marked in the lateral view (I). Myo7a (green) was used to label HCs, and DAPI (blue) was used to label nuclei. *p < 0.05, **p < 0.01, ***p < 0.001. Scale bar, 20 μm.

*Lgr5*‐EGFP‐IRES‐CreERT2 mice (same as *Lgr5* ^*CreER/+*^, Stock #008875) [12, 14] and *Rosa26‐tdTomato* mice (Stock #007914) [12, 62] were used in this study. *Net1*‐floxp mice were constructed by Biocytogen (Beijing, China). CRISPR/Cas9 technology was used to insert the targeting vector, which contained two loxp sites surrounding the STOP sequence and the Net1 coding region with a 3 × HA tag, into the Hipp11 (H11) locus of C57BL/6J mice (Figure 1C). We performed all procedures in compliance with the approved program of the Southeast University Animal Care and Use Committee (No.20210302031) and made every effort to prevent animal suffering.

### 2.2 Genotyping PCR

For mouse genotyping, genomic DNA was extracted by incubating tail tips for 1 h at 98°C in 180 μL (50 mM) NaOH solution, and this was terminated by adding 20 μL Tris‐HCl (1 M, pH = 8). The following primer sequences were utilised for genotyping: *Lgr5*‐Forward: 5′‐CTG CTC TCT GCT CCC AGT CT‐3′; *Lgr5*‐wild type (WT)‐Reverse: 5′‐ATA CCC CAT CCC TTT TGA GC‐3′; *Lgr5*‐mutant (MUT)‐Reverse: 5′‐GAA CTT CAG GGT CAG CTT GC‐3′. *tdTomato*‐WT‐Forward: 5′‐AAG GGA GCT GCA GTG GAG T‐3′; *tdTomato*‐WT‐Reverse: 5′‐CCG AAA ATC TGT GGG AAG TC‐3′; *tdTomato*‐MUT‐Forward: 5′‐GGC ATT AAA GCA GCGT ATC‐3′; *tdTomato*‐MUT‐Reverse: 5′‐CTG TTC CTG TAC GGC ATG G‐3′. *Net1*‐WT‐Forward: 5’‐CAA GCT AAT TTG ACC AGT CAT TGG A‐3′; *Net1*‐WT‐Reverse: 5′‐GCA GAC ACC CAG GAT AAG TG‐3′. *Net1*‐MUT‐Forward: 5’‐GCA TCG ATA CCG TCG ACC TC‐3′. The PCR reaction system was as follows: 2 μL primer mixture, 10 μL 2× Green Taq mix (P131, Vazyme), 3 μL DNA and ddH_2_O up to 20 μL. The PCR reaction conditions were denaturing at 94°C for 5 min followed by 30 cycles of denaturing at 94°C for 30 s, annealing at 58°C for 45 s and extending at 72°C for 45 s.

### 2.3 In Vivo cOE of *Net1* in Lgr5^+^ Progenitors in the Mouse Cochlea

*Net1* ^*loxp/loxp*^ mice were crossed with *Lgr5* ^*CreER/+*^ mice to obtain *Lgr5* ^*CreER/+*^ *Net1* ^*loxp/+*^ and *Lgr5* ^*CreER/+*^ *Net1* ^*loxp/loxp*^ mice. Tamoxifen (T5648, Sigma, 0.075 mg/g body weight) was given intraperitoneally (I.P.) to these mice on postnatal day (P)0‐P1 to activate the Cre recombinase and thus cOE *Net1* in Lgr5^+^ progenitors. The same tamoxifen treatment was also administered to control mice.

### 2.4 RNA Extraction and Real‐Time Fluorescence Quantitative PCR

Dissected mouse cochleae were extracted for total RNA with Trizol Reagent (15596–018, Invitrogen). RNA concentrations were determined and then reverse transcribed to cDNA by using a reverse transcription kit (K1622, Thermo Scientific). Control and experimental cDNA were diluted to the same concentration, and real‐time fluorescence PCR (qPCR) was performed by using 2× Taq Pro‐Universal SYBR qPCR Master Mix Kit (Q712, Vazyme). We used *Gapdh* as the housekeeping gene for mRNA expression normalisation and quantified the gene expression by using the 2^*−∆∆C*^_*T*_ method. Sequences of the qPCR primers are listed in Table S1.

### 2.5 Western Blot

The mouse cochleae were dissected in cold HBSS and lysed in RIPA lysis buffer (P0013C, Beyotime) pre‐mixed with Protease Inhibitor Cocktail (04693132001, Roche) to extract total protein. After grinding and centrifugation, the protein samples were mixed with loading buffer (LT101, Epizyme) and boiled at 98°C for 20 min. Following the separation of protein samples by 10% PAGE, which was performed using the PAGE Gel Rapid Preparation Kit (PG112, Epizyme), the samples were transferred to a PVDF membrane (ISEQ00010, Millipore). The membranes were then blocked with 5% (vol/vol) skimmed milk in 1× TBST (1× Tris buffer solution containing 0.1% Tween‐20) for 1 h at room temperature (RT) and incubated overnight at 4°C with Net1 antibody (sc‐271,941, Santa Cruz) or β‐actin antibody (ab119716, Abcam). The membrane was finally incubated with peroxidase‐conjugated goat anti‐mouse IgG‐HRP and goat anti‐rabbit IgG‐HRP antibodies (M21001; M21002, Abmart) for 1 h at RT after washing three times (10 min each time) with 1× TBST. The target protein bands were imaged on a Tanon‐5200 imaging system (Tanon, China) using the SuperSignal West Dura Persistence Substrate Kit (34,075, Thermo Scientific).

### 2.6 Immunofluorescent Staining

P7 mouse cochleae were dissected and fixed for 2 h in 4% paraformaldehyde (P0099, Beyotime Biotechnology) at RT. The cochleae were then dissected in cold HBSS buffer, attached to a glass coverslip coated with Cell‐Tak (354,240, Corning) and blocked for 1 h with blocking solution (0.5% Triton‐X100, 1% BSA and 10% donkey serum in PBS) at RT. Primary antibodies diluted in PBST‐1 (0.1% Triton‐X100, 1% BSA and 5% donkey serum in PBS) were incubated with the cochleae overnight at 4°C. The next day the cochleae were stained with fluorescence‐conjugated secondary antibody (A32731, A21432, A32727, Invitrogen) and DAPI (C0060, Solarbio) for 1 h in the dark and then washed three times (5 min each time) with 0.1% Triton‐X100 in PBS. Finally, 6 μL of antifade fluorescence mounting medium (S3023, DAKO) was added for mounting. The following primary antibodies were used: Myosin7a (#25–6790, Proteus Bioscience, 1:1000 dilution in PBST‐1), Sox2 (AF2018, R&D system, 1:400 dilution in PBST‐1) and HA (M20003, Abmart, 1:400 dilution in PBST‐1). All immunofluorescent staining samples were imaged on a laser confocal microscopy (LSM 900, Zeiss).

### 2.7 Labelling Proliferating Cells With 5‐Ethynyl‐2′‐Deoxyuridine (EdU)

Tamoxifen was injected into *Lgr5* ^*CreER/+*^ *Net1* ^*loxp/loxp*^ and *Net1* ^*loxp/loxp*^ mice at P0‐P1, and then the cell proliferation marker EdU was injected once a day from P3 to P5. The mouse cochleae were dissected at P7 and stained with the Alexa Fluor 555 reagent of the BeyoClick EdU Cell Proliferation Kit (C0075S, Beyotime) for 30 min to label the EdU^+^ proliferating cells with red fluorescence. The basilar membranes (BMs) of P3 WT mouse cochlea were dissected and cultured with proliferation medium for 3 days in vitro, and 10 μM EdU was added to the culture medium for the whole culture period. Samples were collected for EdU staining as a positive control for the EdU assay. The proliferation medium is composed of DMEM/F12 (12,634,010, Thermo Fisher), N2 (1%, 17,502,001, Thermo Fisher), B27 (2%, 12,587,010, Thermo Fisher), EGF (20 ng/mL, E9644, Sigma), β‐FGF (10 ng/mL, 100‐18C, Peprotech), CHIR99021 (3 μM, SML1046, Sigma‐Aldrich), valproic acid (1 mM, P4543, Sigma‐Aldrich), 616,452 (2 μM, 446,859–33‐2, Sigma‐Aldrich) and 0.1% ampicillin (A610028‐0025, Sangon Biotech). EdU^+^/Sox2^+^ cells were observed by laser confocal microscope (LSM 900, Zeiss) and counted.

### 2.8 Lineage Tracing of Lgr5^+^ Progenitors in *Lgr5* ^*CreER*^ ^*/+*^ *Net1^loxp/loxp^tdtomato* Mouse Cochleae In Vivo

*Lgr5* ^*CreER/+*^ *Net1* ^*loxp/loxp*^ mice were crossed with *Net1* ^*loxp/loxp*^ *tdTomato* mice to obtain *Lgr5* ^*CreER/+*^ *Net1* ^*loxp/loxp*^ *tdTomato* mice. Lineage tracing of Lgr5^+^ progenitors in the cochlea was performed using these triple‐positive mice. *Lgr5* ^*CreER/+*^ *Net1* ^*loxp/loxp*^ *tdTomato* mice and *Lgr5* ^*CreER/+*^ *tdTomato* mice were I.P. injected with tamoxifen at P0‐P1 to cOE *Net1*, and cochleae were dissected at P7 for staining and for statistical analysis of newly regenerated tdTomato^+^ HCs.

### 2.9 Statistical Analysis

Each group of experiments had three or more sets of repetitions under the same conditions. The n in the figure represents the number of qPCR repetitions or mouse cochleae used. We analysed significance by using a two‐tailed unpaired Student's t‐test or two‐way ANOVA followed by a Bonferroni post‐test when comparing two groups or more than two groups, respectively. Data were processed with GraphPad Prism 8 software and presented as the mean **±** standard error of the mean (S.E.M). A *p*‐value < 0.05 was considered statistically significant.

## 3 Results

### 3.1 Expression Pattern of Net1 in the Mouse Cochlea and cOE of *Net1* in Lgr5^+^ Progenitors of the Neonatal Mouse Cochlea

We first detected the cochlear expression of Net1 at different developmental time points of WT mice in both the embryonic and postnatal stages. We observed that Net1 is expressed in the cochlea of both embryonic and postnatal mice, with both its mRNA and protein expression levels initially increasing and then decreasing after birth (Figures 1A,B and S1A), suggesting that Net1 might have a specific role in the development of cochlear HCs. The *Net1* floxp mice were constructed as illustrated in Figure 1C and genotyped (Figure 1D). The P0‐P1 *Lgr5* ^*CreER/+*^ *Net1* ^*loxp/loxp*^ mice were I.P. injected with tamoxifen to overexpress *Net1* in Lgr5^+^ progenitors (Figure 1E,F). The *Net1* ^*loxp/loxp*^ mice served as controls. We detected the expression specificity of cOE of *Net1* in the cochlear BM of *Lgr5* ^*CreER/+*^ *Net1* ^*loxp/loxp*^ mice compared with other parts of the cochlea, for example modiolus (M) and spiral ligament (SL). And we found that the expression of *Net1* is only upregulated in cochlear BM of *Lgr5* ^*CreER/+*^ *Net1* ^*loxp/loxp*^ mice compared with the control mice, rather than in M and SL (Figure S1B). In addition, the mRNA level of *Net1* in cochlear BM was detected in *Net1* ^*loxp/+*^ mice, *Lgr5* ^*CreER/+*^ *Net1* ^*loxp/+*^ mice and *Lgr5* ^*CreER/+*^ *Net1* ^*loxp/loxp*^ mice (Figure 1H), and the results showed dose‐dependent expression of *Net1* in these three transgenic mice. And cOE of *Net1* was also observed in Lgr5^+^ progenitors by HA tag staining of *Lgr5* ^*CreER/+*^ *Net1* ^*loxp/loxp*^ mice cochlea (Figure 1I). These results showed that *Net1* is specifically overexpressed in the Lgr5^+^ progenitors in cochlear BM.

### 3.2 A Significant Increase in Ectopic OHCs and IHCs was Observed in the Cochlea of *Net1* cOE *Lgr5* ^*CreER*^ ^*/+*^ *Net1* ^*loxp/loxp*^ Mice

**FIGURE 2:**
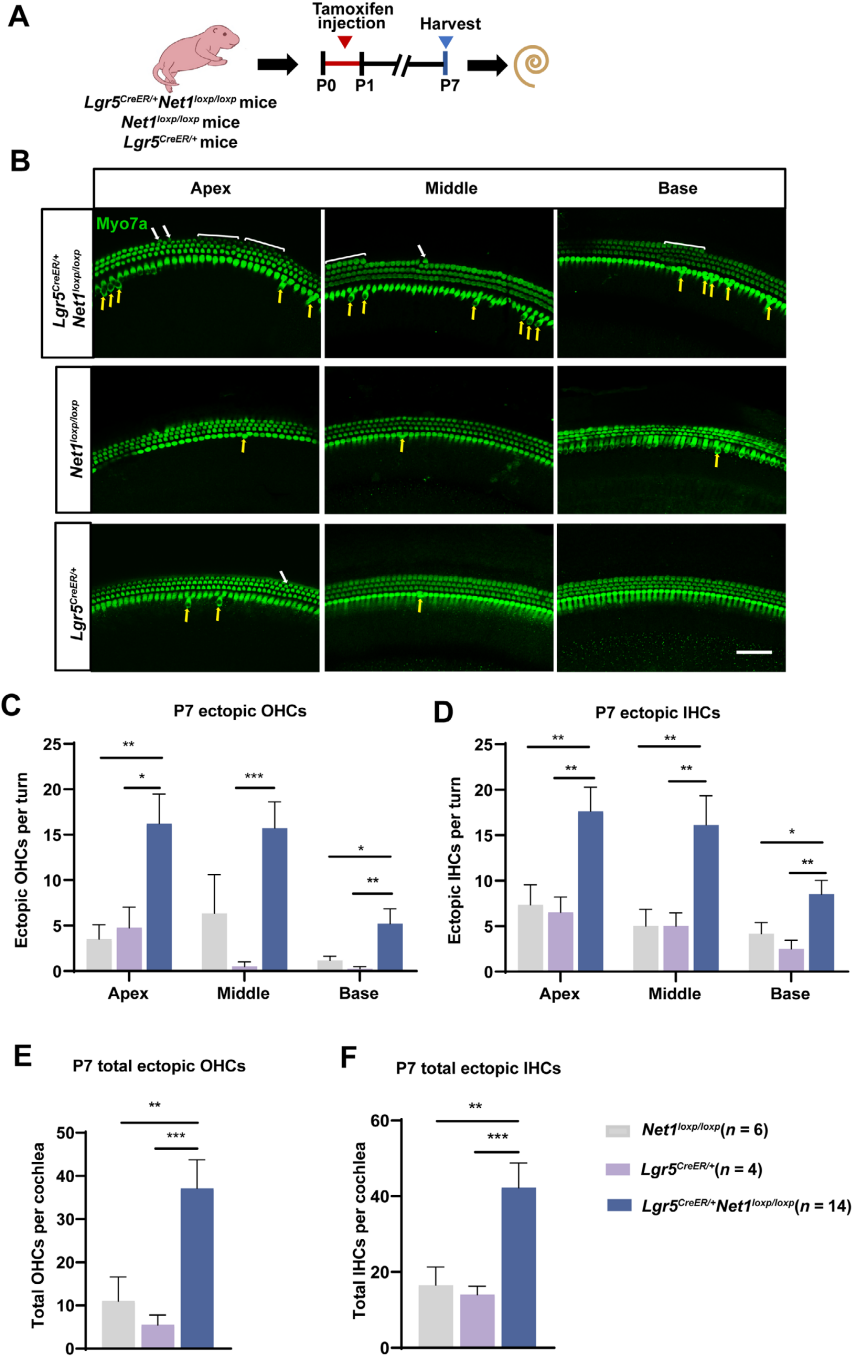
Ectopic HCs were significantly increased in *Net1* homozygous cOE *Lgr5* ^*CreER/+*^ *Net1* ^*loxp/loxp*^ mice. (A) Tamoxifen was I.P. injected into P0‐P1 *Lgr5* ^*CreER/+*^ *Net1* ^*loxp/loxp*^ mice to activate Cre recombinase and subsequently overexpress *Net1* in Lgr5^+^ progenitors. (B) Ectopic OHCs (white arrows and white brackets) and IHCs (yellow arrows) were seen in the apical, middle and basal turns of the P7 *Lgr5* ^*CreER/+*^ *Net1* ^*loxp/loxp*^ mouse cochlea. *Lgr5* ^*CreER/+*^ and *Net1* ^*loxp/loxp*^ mice were used as controls. Myo7a (green) was used as the HC marker. Scale bar, 50 μm. (C–F) Quantification of the ectopic OHCs (C) and IHCs (D) per turn and the total ectopic OHCs (E) and total ectopic IHCs (F) per cochlea. ‘*n*’ refers to the number of mice. *p < 0.05, **p < 0.01, ***p < 0.001.

To study the effect of *Net1* cOE in the neonatal mouse cochlea, tamoxifen was I.P. injected into *Lgr5* ^*CreER/+*^ *Net1* ^*loxp/loxp*^ mice at P0‐P1, and the cochleae were dissected for staining at P7 (Figure 2A). The *Net1* ^*loxp/loxp*^ mice and *Lgr5* ^*CreER/+*^ mice were treated in the same way as controls. An extensive number of ectopic OHCs and IHCs was observed in P7 *Lgr5* ^*CreER/+*^ *Net1* ^*loxp/loxp*^ mice compared with control mice (Figure 2B). The number of ectopic HCs increased from the basal to apical turns, which is perhaps related to the HC regeneration ability increasing in a basal‐apical gradient [9, 47]. We quantified the HCs in the apical, middle and basal turns of the cochlea and found that the number of ectopic IHCs and OHCs both increased significantly in each turn (Figure 2C,D). Additionally, the total ectopic OHCs and IHCs in each cochlea were also significantly increased in *Lgr5* ^*CreER/+*^ *Net1* ^*loxp/loxp*^ mice (37.14 ± 6.58 ectopic OHCs per cochlea, 42.29 ± 6.48 ectopic IHCs per cochlea) compared to *Net1* ^*loxp/loxp*^ mice (11.00 ± 5.59 ectopic OHCs per cochlea, 16.50 ± 4.81 ectopic IHCs per cochlea) and *Lgr5* ^*CreER/+*^ mice (5.50 ± 2.33 ectopic OHCs per cochlea, 14.00 ± 2.27 ectopic IHCs per cochlea) (Figure 2E,F and Table S2). These findings demonstrate that *Net1* cOE in Lgr5^+^ progenitors induced the regeneration of OHCs and IHCs in the apical, middle and basal turns of the neonatal mouse cochlea.

### 3.3 HC Regeneration Induced by *Net1* cOE Showed a Dose‐Dependent Effect

**FIGURE 3:**
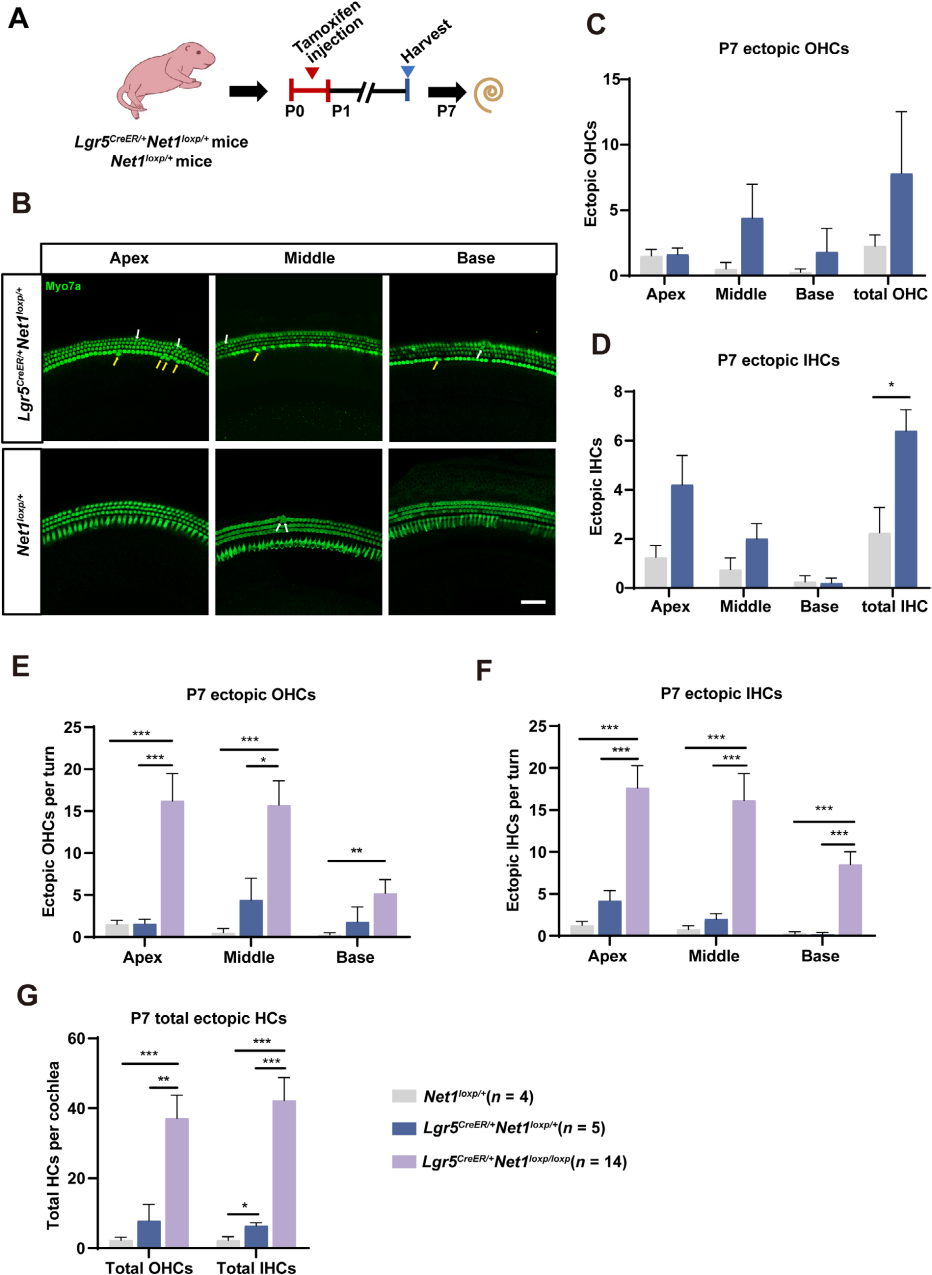
Only ectopic IHCs were slightly but significantly increased in *Net1* heterozygous cOE *Lgr5* ^*CreER/+*^ *Net1* ^*loxp/+*^ mice. (A) *Lgr5* ^*CreER/+*^ *Net1* ^*loxp/+*^ mice and *Net1* ^*loxp/+*^ mice were I.P. injected with tamoxifen at P0‐P1, and the mice were sacrificed at P7. *Net1* ^*loxp/+*^ mice were used as controls. (B) Ectopic OHCs (white arrows) and IHCs (yellow arrows) were seen in the apical, middle and basal turns of *Lgr5* ^*CreER/+*^ *Net1* ^*loxp/+*^ mice. Myo7a (green) was used as the HC marker. Scale bar, 50 μm. (C, D) Quantification of the ectopic OHCs per turn and the total ectopic OHCs per cochlea (C), and quantification of the ectopic IHCs per turn and the total ectopic IHCs per cochlea of *Net1* ^*loxp/+*^ mice and *Lgr5* ^*CreER/+*^ *Net1* ^*loxp/+*^ mice (D). ‘n’ refers to the number of cochleae. (E–G) Quantification of the ectopic OHCs per turn (E) and the ectopic IHCs per turn (F), and quantification of the total ectopic OHCs and IHCs per cochlea of *Net1* ^*loxp/+*^ mice, *Lgr5* ^*CreER/+*^ *Net1* ^*loxp/+*^ mice and *Lgr5* ^*CreER/+*^ *Net1* ^*loxp/loxp*^ mice (G). ‘n’ refers to the number of cochleae. *p < 0.05. **p < 0.01, ***p < 0.001.

Considering that the roles of some genes often show a dose‐dependent effect, we also studied the phenotype of *Net1* cOE *Lgr5* ^*CreER/+*^ *Net1* ^*loxp/+*^ mice. Tamoxifen was I.P. injected into *Lgr5* ^*CreER/+*^ *Net1* ^*loxp/+*^ mice at P0‐P1, and the cochleae were dissected at P7 to determine the number of HCs (Figure 3A,B). Quantitative results suggested that the number of total ectopic IHCs in *Lgr5* ^*CreER/+*^ *Net1* ^*loxp/+*^ mice (6.40 ± 0.87 ectopic IHCs per cochlea) was slightly but significantly increased compared with control *Net1* ^*l*oxp/+^ mice (2.25 ± 1.03 ectopic IHCs per cochlea), while there was no significant difference in the apical turn, middle turn or basal turn (Figure 3D, Table S3). In addition, the number of ectopic OHCs was not significantly different from that of control mice (Figure 3C). Furthermore, the number of ectopic OHCs and IHCs in the apical, middle and basal turns was significantly increased in *Lgr5* ^*CreER/+*^ *Net1* ^*loxp/loxp*^ mice than in *Lgr5* ^*CreER/+*^ *Net1* ^*loxp/+*^ mice and *Net1* ^*l*oxp/+^ mice (Figure 3E–G and Table S3). These results showed that the number of ectopic OHCs and IHCs increased along with the increase of *Net1* expression level, which demonstrates that *Net1* induces HC regeneration in a dose‐dependent manner.

### 3.4 The Ectopic HCs Arise from Direct Trans‐Differentiation of Lgr5^+^ Progenitors in *Lgr5* ^*CreER*^ ^*/+*^ *Net1* ^*loxp/*^ ^*loxp*^ *tdTomato* Mouse Cochleae

**FIGURE 4:**
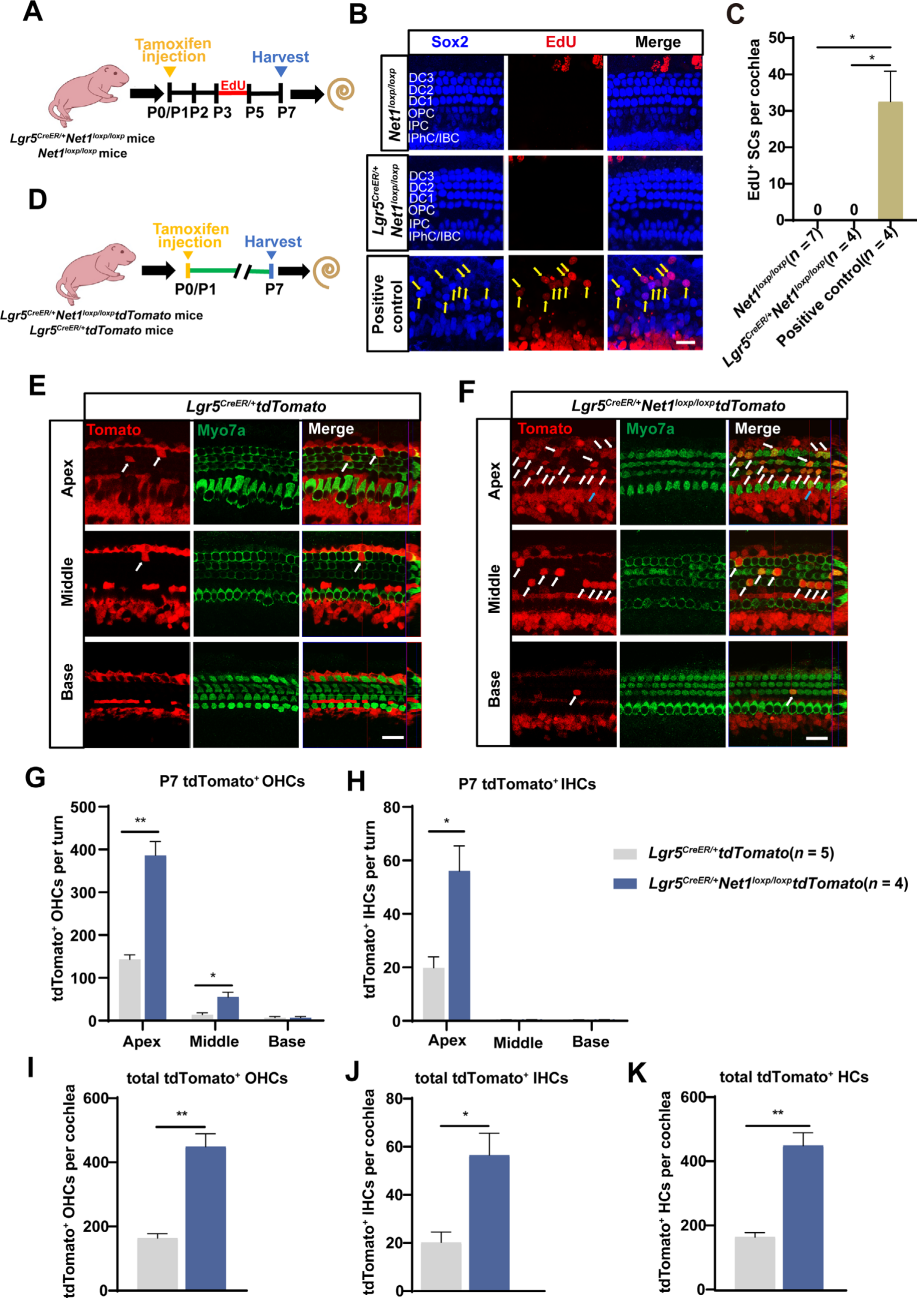
EdU assay and lineage tracing of Lgr5^+^ progenitors. (A) Tamoxifen was injected into *Lgr5* ^*CreER/+*^ *Net1* ^*loxp/loxp*^ mice to conditionally overexpress *Net1* in Lgr5^+^ progenitors. EdU (50 mg/kg body weight) was injected at P3‐P5 to label proliferating cells. (B) EdU was stained (red) in *Net1* ^*loxp/loxp*^ mice, *Lgr5* ^*CreER/+*^ *Net1* ^*loxp/loxp*^ mice and positive control cochlea. Sox2 (blue) was used as the SC marker. EdU^+^/Sox2^+^ SCs are indicated by yellow arrows. Scale bar, 20 μm. (C) Quantification of EdU^+^ SCs per cochlea. ‘n’ represents the number of mice. (D) Tamoxifen was injected into *Lgr5* ^*CreER/+*^ *Net1* ^*loxp/loxp*^ *tdTomato* and *Lgr5* ^*CreER/+*^ *tdTomato* mice at P0‐P1 to lineage trace Lgr5^+^ progenitors at P7. (E, F) Lineage tracing images of tdTomato^+^ HCs in the cochlea of *Lgr5* ^*CreER/+*^ *tdTomato* mice (E) and *Lgr5* ^*CreER/+*^ *Net1* ^*loxp/loxp*^ *tdTomato* mice (F). Myo7a (green) was used as the HC marker. tdTomato^+^ OHCs are indicated by white arrows, and tdTomato^+^ IHCs are indicated by blue arrows. Scale bar, 20 μm. (G–K) Quantification of tdTomato^+^ OHCs (G) and IHCs (H) per turn, total tdTomato^+^ OHCs (I) and IHCs (J) per cochlea, and total tdTomato^+^ HCs (K). ‘n’ represents the number of mice. *p < 0.05, **p < 0.01, ***p < 0.001.

Numerous studies have shown that HCs derive from progenitors or SCs via mitotic regeneration or via direct trans‐differentiation [12]. To determine the mechanism through which *Net1* cOE promotes HC regeneration, we performed an EdU assay and a lineage‐tracing assay. We first injected tamoxifen into *Lgr5* ^*CreER/+*^ *Net1* ^*loxp/loxp*^ mice at P0‐P1, followed by EdU injection (5 mg/g body weight) to label proliferating cells for three successive days from P3 to P5 (Figure 4A). We observed no EdU^+^/Sox2^+^ SCs in the *Lgr5* ^*CreER/+*^ *Net1* ^*loxp/loxp*^ mouse cochlea or in the *Net1* ^*loxp/loxp*^ mice, compared with a significant large number of EdU^+^ SCs in the positive control cultured with proliferation medium (Figure 4B,C). These results suggested that the ectopic HCs were probably not regenerated from Lgr5^+^ progenitors via mitotic generation in vivo.

Next, *Lgr5* ^*CreER/+*^ *Net1* ^*loxp/loxp*^ mice were crossed with *Rosa26‐tdTomato* mice to obtain *Lgr5* ^*CreER/+*^ *Net1* ^*loxp/loxp*^ *Rosa26‐tdTomato* triple‐positive mice for Lgr5^+^ progenitors lineage tracing. Tamoxifen was I.P. injected in both triple‐positive mice and control mice at P0–P1, and mouse cochleae were dissected for staining at P7 (Figure 4D). We observed a great many more tdTomato^+^ HCs in *Lgr5* ^*CreER/+*^ *Net1* ^*loxp/loxp*^ *tdTomato* mice (505.00 ± 47.32 tdTomato^+^ HCs per cochlea) compared to control mice (184.00 ± 14.55 tdTomato^+^ HCs per cochlea) (Figure 4E,F,K). The statistical analysis also showed a significantly increased number of tdTomato^+^ OHCs (448.5 ± 40.33 total tdTomato^+^ OHCs per cochlea) in the apical turn and middle turn, while a statistically significant increase in tdTomato^+^ IHCs (56.50 ± 9.15 total tdTomato^+^ IHCs per cochlea) was only seen in the apical turn of the P7 triple‐positive mouse cochlea (Figure 4G–J and Table S4). These results indicate that, instead of mitotic HC regeneration, the numerous ectopic HCs in *Lgr5* ^*CreER/+*^ *Net1* ^*loxp/loxp*^ mice probably arose by direct trans‐differentiation from Lgr5^+^ progenitors.

### 3.5 Genes and Pathways Involved in *Lgr5* ^*CreER*^ ^*/+*^ *Net1* ^*loxp/loxp*^ Mice Induced HC Regeneration

**FIGURE 5:**
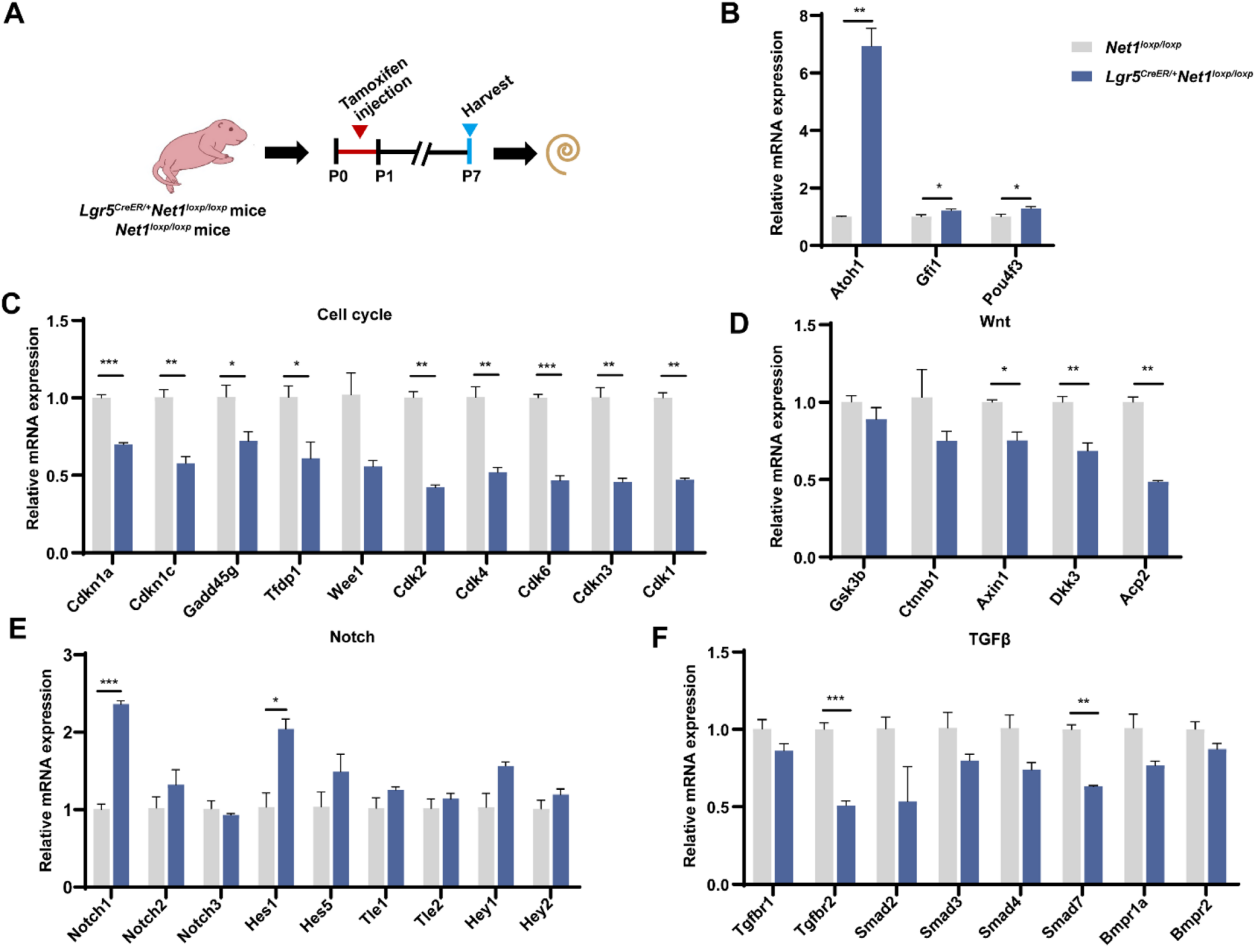
Quantification of the expression of related genes and signalling pathways in *Lgr5* ^*CreER/+*^ *Net1* ^*loxp/loxp*^ mice. (A) *Lgr5* ^*CreER/+*^ *Net1* ^*loxp/loxp*^ mice and *Net1* ^*loxp/loxp*^ mice were injected with tamoxifen at P0‐P1, and the RNA of the mouse cochlea was extracted at P7. *Net1* ^*loxp/loxp*^ mice were used as controls. (B–F) Relative mRNA expression of transcription factors related to HC regeneration (B), cell cycle (C), Wnt signalling (D), Notch signalling (E) and TGFβ signalling pathways (F). Three independent qPCR experiments were performed. *p < 0.05, **p < 0.01, ***p < 0.001.

To study the possible mechanisms by which *Net1* enhances HC regeneration, the mRNA of the *Lgr5* ^*CreER/+*^ *Net1* ^*loxp/loxp*^ mouse cochlea was extracted, and the expression levels of some important genes and signalling pathways related to HC regeneration were measured by qPCR (Figure 5A). We found that the expression levels of the transcription factors *Atoh1*, *Gfi1* and *Pou4f3*, which are involved in HC differentiation and maturation [63, 64, 65], were all significantly up‐regulated in *Lgr5* ^*CreER/+*^ *Net1* ^*loxp/loxp*^ mice, with *Atoh1* expression increasing the most (about a 6‐fold increase) (Figure 5B). Considering that *Net1* has been reported to be involved in regulating the Wnt, Notch and TGFβ signalling pathways and regulating the cell cycle [65, 66, 67], which are also closely related to the process of HC regeneration, we analysed gene expression in these pathways by qPCR. The cell cycle inhibitors *Cdkn1a*, *Cdkn1c*, *Cdkn3* and *Gadd45g* were all downregulated, and the cell cycle‐dependent kinases *Cdk1/2/4/6* and *Tfdp1* were also downregulated in *Lgr5* ^*CreER/+*^ *Net1* ^*loxp/loxp*^ mice (Figure 5C), which suggests that the cell cycle may not be the main pathway regulated by *Net1* cOE in the process of HC regeneration. The expression of negative regulators of the Wnt signalling pathway – *Axin2*, *Acp2* and *Dkk3* – were slightly downregulated, while the expression of *GSK3β* and *Ctnnb1*, the main regulator and effector of the Wnt signalling pathway, respectively, was not changed significantly (Figure 5D). Only *Tgfbr2* and *Smad7* in the TGFβ pathway were down‐regulated (Figure 5E), and only *Notch1* and *Hes1* in the Notch pathway were up‐regulated (Figure 5F). Because the expression of critical genes in the Wnt, TGFβ and Notch pathways was not changed significantly, *Net1* cOE may likely not facilitate HC regeneration through these signalling pathways. In summary, all these results suggest that *Net1* cOE in Lgr5^+^ progenitors induces ectopic HC generation by activating *Atoh1*, *Gfi1* and *Pou4f3*, especially *Atoh1*.

## 4 Discussion

Sensory HCs convert mechanical acoustic impulses into electrical nerve signals, which are transmitted to the auditory cortex to ultimately produce hearing. In the mammalian cochlea, HC damage cannot be replenished by regeneration, meaning that HC damage in mammals results in permanent hearing loss [7, 8]. Thus, the identification of new genes that promote HC regeneration has been a primary focus of hearing recovery research. Lgr5^+^ SCs in the neonatal cochlea behave like progenitors that can regenerate HCs through either mitosis or direct trans‐differentiation [12, 13, 14]. We previously conducted a joint analysis of three transcriptomes and showed that some genes might regulate HC regeneration, including *Net1* (unpublished). In this study, we used an *in vivo* mouse model and found that *Net1* cOE facilitated HC regeneration through the direct trans‐differentiation of Lgr5^+^ progenitors.

Net1, a RhoA subfamily guanine‐nucleotide exchange factor, is primarily involved in the proliferation, migration and differentiation of several types of cancer cells, including gastric, lung and breast cancer [56, 57, 59]. In this study, we found that in *Net1* cOE *Lgr5* ^*CreER/+*^ *Net1* ^*loxp/loxp*^ mice, the number of ectopic OHCs and IHCs increased from the basal turn to the apical turn. This is consistent with a gradual increase in the ability of Lgr5^+^ progenitors to proliferate and differentiate into HCs from the basal to the apical turn [37, 48, 68]. We then compared the ectopic OHCs and IHCs numbers of *Net1* cOE *Lgr5* ^*CreER/+*^ *Net1* ^*loxp/loxp*^ mice (37.14 ± 6.58 ectopic OHCs per cochlea, 42.29 ± 6.48 ectopic IHCs per cochlea) and *Lgr5* ^*CreER/+*^ *Net1* ^*loxp/+*^ mice (7.80 ± 4.73 ectopic OHCs per cochlea, 6.40 ± 0.87 ectopic IHCs per cochlea) and found that the total OHCs and total IHCs number of *Lgr5* ^*CreER/+*^ *Net1* ^*loxp/loxp*^ mice increased significantly compared with *Lgr5* ^*CreER/+*^ *Net1* ^*loxp/+*^ mice (Figure 3E–G and Table S3), indicating that *Net1* overexpression promotes HC regeneration in a dose‐dependent manner.

We further identified the way in which ectopic HCs are derived from Lgr5^+^ progenitors by labelling proliferating cells with EdU and lineage tracing the Lgr5^+^ progenitors. We failed to observe any EdU^+^/Sox2^+^ SCs in the EdU assay, while we observed a significant increase in myo7a^+^/tdTomato^+^ cells in *Lgr5* ^*CreER/+*^ *Net1* ^*loxp/loxp*^ *tdTomato* mice compared with control mice in the lineage tracing assay, suggesting that ectopic HCs derive from direct trans‐differentiation from Lgr5^+^ progenitors rather than through mitotic regeneration. We also checked the expression of cell cycle‐related genes and found that both cell cycle inhibitors and cyclin‐dependent kinases were down‐regulated, suggesting that *Net1* cOE might not affect the cell cycle, which is consistent with the EdU assay results. We further explored other possible mechanisms by which *Net1* cOE might induce the direct trans‐differentiation of Lgr5^+^ progenitors into HCs and checked the expression of several signalling pathways such as Wnt, TGFβ and Notch, which are reported to have crosstalk with *Net1* [61, 65, 67, 69] and are also strongly connected to the differentiation of Lgr5^+^ progenitors into HCs [23, 70, 71]. The results showed that most of the genes in the Wnt, TGFβ and Notch pathways were not significantly changed, suggesting that the promotion of Lgr5^+^ progenitors into HCs by *Net1* is not strongly associated with these pathways. Thus, further exploration of the specific pathway by which *Net1* is involved in HC regeneration will be required in the future.

The transcription factor Atoh1 (also known as Math1) is a basic helix‐loop‐helix protein expressed in sensory epithelial cells of the inner ear, and *Atoh1* deletion in embryonic mice results in the complete loss of vestibular and cochlear HCs [72]. Ectopic expression of *Atoh1* in neonatal or adult mouse otocysts drives cochlear progenitor cells towards a HC fate, thus inducing the generation of functional sensory HCs [24, 45, 73]. The transcription factors *Gfi1* and *Pou4f3* are required for HC survival and maturation, and *Gfi1*‐null mice and *Pou4f3*‐null mice show HC death and failure to mature properly [64, 74]. In addition, Gfi1 and Pou4f3 are downstream targets of Atoh1 during development [75, 76], and both Gfi1 and Pou4f3 have been shown to enhance Atoh1‐induced SC conversion to HCs [41, 77]. In the present study, we found an approximately 6‐fold increase in the expression of *Atoh1* in the cochlea of *Lgr5* ^*CreER/+*^ *Net1* ^*loxp/loxp*^ mice compared to control mice, along with significant upregulation of *Gfi1* and *Pou4f3*. These results suggest that the activation of *Atoh1*, *Gfi1* and *Pou4f3* are the likely mechanisms involved in *Net1*‐induced trans‐differentiation of Lgr5^+^ progenitors into HCs.

In conclusion, we conditionally overexpressed *Net1* in Lgr5^+^ progenitors of the neonatal mouse cochlea and observed a significantly increased number of ectopic HCs and tdTomato^+^ HCs compared to control mice. We further found that *Net1* cOE leads to supernumerary HC production by inducing the direct trans‐differentiation of Lgr5^+^ progenitors. The expression of the transcription factors *Atoh1*, *Gfi1* and *Pou4f3* was greatly increased, especially *Atoh1*, which may play a crucial role in regulating ectopic HC regeneration in the cochlea in *Lgr5* ^*CreER/+*^ *Net1* ^*loxp/loxp*^ mice. Additionally, some specific signalling pathway may also participate in the process through which *Net1* regulates HC regeneration, and this needs further exploration. In summary, this study offers new evidence for the role of *Net1* in cochlear HC regeneration in neonatal mice.

## Author Contributions

S.Z. conceived and designed the experiments. Y.L., Q.Z., W.T., and Y.W. performed the experiments. Y.L., S.Z., L.W., H.X., X.T., M.D., and Z.Y. analysed the data. S.Z., Y.L., and R.C. wrote and edited the manuscript. All authors read and approved the final manuscript.

## Conflicts of Interest

The authors declare no conflicts of interest.

## Supporting information


Data S1.

## Data Availability

The data that support the findings of this study are available from the corresponding author upon reasonable request.
