# Supplementary material for: Conditional Overexpression of Net1 Enhances the Trans‐Differentiation of Lgr5+ Progenitors into Hair Cells in the Neonatal Mouse Cochlea
Source: Cell Prolif. 2024 Dec 15;58(4):e13787. doi: 10.1111/cpr.13787 (PMC11969244; doi:10.1111/cpr.13787)
Supplement: Supplementary file 1 — Data S1. [file CPR-58-e13787-s001.docx]

**Fig. S1**


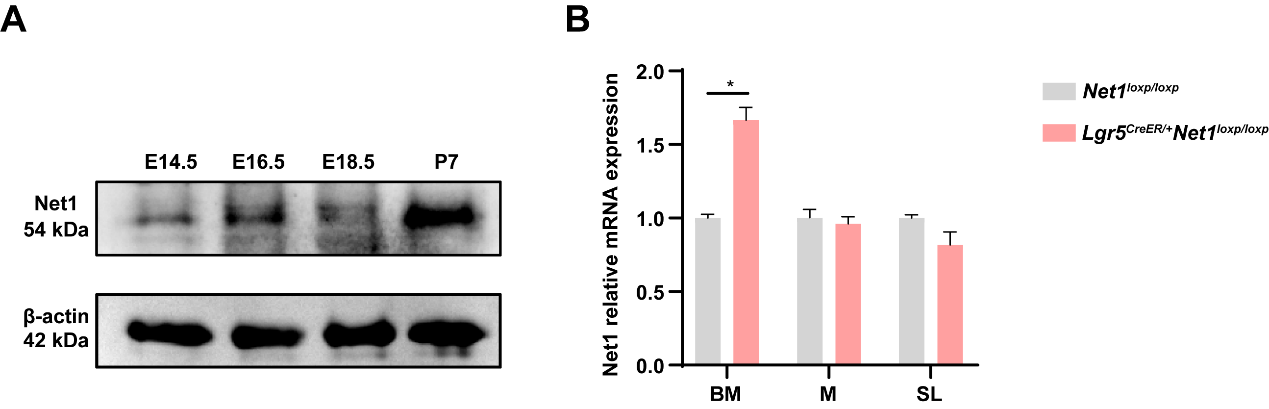


Fig. S1 **(A)** Net1 protein expression at E14.5/E16.5/E18.5 in the mouse cochlea was detected by Western blotting. **(B)** *Net1* overexpression in the mouse cochlear basilar membrane (BM), modiolus (M) and spiral ligament (SL) was verified by real-time qPCR. **P* < 0.05.

Supplementary Table 1

The primer sequences for qPCR.

| Gene | Forward (5’-3’) | Reverse (5’-3’) |
| --- | --- | --- |
| *Net1* | CGGCGAACGAGAGATGCTC | CTCCTTCAAATCAAGGCTGCTA |
| *Gapdh* | AGGTCGGTGTGAACGGATTTG | TGTAGACCATGTAGTTGAGGTCA |
| *Atoh1* | GAGTGGGCTGAGGTAAAAGAGT | GGTCGGTGCTATCCAGGAG |
| *Gfi1* | AGAAGGCGCACAGCTATCAC | GGCTCCATTTTCGACTCGC |
| *Pou4f3* | ATGCGCCGAGTTTGTCTCC | GGGCTTGAACGGATGATTCTTG |
| *GSK3β* | TGGCAGCAAGGTAACCACAG | CGGTTCTTAAATCGCTTGTCCTG |
| *Ctnnb1* | ATGGAGCCGGACAGAAAAGC | CTTGCCACTCAGGGAAGGA |
| *Axin1* | CTCCAAGCAGAGGACAAAATCA | GGATGGGTTCCCCACAGAAATA |
| *Dkk3* | CTCGGGGGTATTTTGCTGTGT | TCCTCCTGAGGGTAGTTGAGA |
| *Acp2* | CACACAGTTTGACCATCGTGA | GTGGACGAGGTTGCGTAGC |
| *Cdkn1a* | CCTGGTGATGTCCGACCTG | CCATGAGCGCATCGCAATC |
| *Cdkn1c* | CGAGGAGCAGGACGAGAATC | GAAGAAGTCGTTCGCATTGGC |
| *Gadd45g* | GGGAAAGCACTGCACGAACT | AGCACGCAAAAGGTCACATTG |
| *Tfdp1* | TTGAAGCCAACGGAGAACTAAAG | TGGACTGTCCGAAGGTTTTTG |
| *Wee1* | GTCGCCCGTCAAATCACCTT | GAGCCGGAATCAATAACTCGC |
| *Cdk2* | CCTGCTTATCAATGCAGAGGG | GTGCTGGGTACACACTAGGTG |
| *Cdk4* | ATGGCTGCCACTCGATATGAA | TCCTCCATTAGGAACTCTCACAC |
| *Cdk6* | GGCGTACCCACAGAAACCATA | AGGTAAGGGCCATCTGAAAACT |
| *Cdkn3* | TCCTCTGCCGTCAGGACAAA | GTAGTACCCTTGAAGTGTGGAAG |
| *Cdk1* | AGAAGGTACTTACGGTGTGGT | GAGAGATTTCCCGAATTGCAGT |
| *Tgfbr1* | TCCCAACTACAGGACCTTTTTCA | GCAGTGGTAAACCTGATCCAGA |
| *Tgfbr2* | CCGCTGCATATCGTCCTGTG | AGTGGATGGATGGTCCTATTACA |
| *Smad2* | ATGTCGTCCATCTTGCCATTC | AACCGTCCTGTTTTCTTTAGCTT |
| *Smad3* | CACGCAGAACGTGAACACC | GGCAGTAGATAACGTGAGGGA |
| *Smad4* | ACACCAACAAGTAACGATGCC | GCAAAGGTTTCACTTTCCCCA |
| *Smad7* | GGCCGGATCTCAGGCATTC | TTGGGTATCTGGAGTAAGGAGG |
| *Bmpr1a* | AACAGCGATGAATGTCTTCGAG | GTCTGGAGGCTGGATTATGGG |
| *Bmpr2* | TTGGGATAGGTGAGAGTCGAAT | TGTTTCACAAGATTGATGTCCCC |
| *Notch1* | CCGTGTAAGAATGCTGGAACG | AGCGACAGATGTATGAAGACTCA |
| *Notch2* | ATGTGGACGAGTGTCTGTTGC | GGAAGCATAGGCACAGTCATC |
| *Notch3* | TGCCAGAGTTCAGTGGTGG | CACAGGCAAATCGGCCATC |
| *Hes1* | CCAGCCAGTGTCAACACGA | AATGCCGGGAGCTATCTTTCT |
| *Hes5* | AGTCCCAAGGAGAAAAACCGA | GCTGTGTTTCAGGTAGCTGAC |
| *Tle1* | CCAGTACCTCTCACGCCTCA | GCCCACTCAGAGCACTAGAC |
| *Tle2* | TGGCTGCCGTAAAGGAAGAC | CTCACTGTCATAAGGCCCTGA |
| *Hey1* | GCGCGGACGAGAATGGAAA | TCAGGTGATCCACAGTCATCTG |
| *Hey2* | AAGCGCCCTTGTGAGGAAAC | GGTAGTTGTCGGTGAATTGGAC |

Supplementary Table 2

Quantification of ectopic HCs in P7 *Lgr5^CreER/+^Net1^loxp/loxp^* mice, *Lgr5^CreER/+^* mice, *Net1^loxp/loxp^* mice. The n represents the number of mice used.

|  | *Lgr5^CreER/+^Net1^loxp/loxp^* (n=14） | *Lgr5^CreER/+^* (n=4) | *Net1^loxp/loxp^* (n=6) |
| --- | --- | --- | --- |
| Ectopic OHCs per turn (Apex) | 16.21 ± 3.25 | 4.75 ± 2.29 | 3.50 ± 1.61 |
| Ectopic OHCs per turn (Middle) | 15.71 ± 2.90 | 0.50 ± 0.50 | 6.33 ± 4.27 |
| Ectopic OHCs per turn (Base) | 5.21 ± 1.64 | 0.25 ± 0.25 | 1.17 ± 0.48 |
| Ectopic IHCs per turn (Apex) | 17.64 ± 2.64 | 6.50 ± 1.71 | 7.33 ± 2.22 |
| Ectopic IHCs per turn (Middle) | 16.14 ± 3.19 | 5.00 ± 1.47 | 5.00 ± 1.86 |
| Ectopic IHCs per turn (Base) | 8.50 ± 1.53 | 2.50 ±0.96 | 4.17 ±1.25 |
| Total ectopic OHCs per cochlea | 37.14 ± 6.58 | 5.50± 2.33 | 11.00 ± 5.59 |
| Total ectopic IHCs per cochlea | 42.29 ± 6.48 | 14.00± 2.27 | 16.50 ± 4.81 |

Supplementary Table 3

Quantification of ectopic HCs in P7 *Lgr5^CreER/+^Net1^loxp/+^*, *Lgr5^CreER/+^Net1^loxp/loxp^* and *Net1^loxp/+^* mice. The n represents the number of mice used.

|  | *Lgr5^CreER/+^Net1^loxp/+^* (n=5） | *Lgr5^CreER/+^Net1^loxp/loxp^* (n=14） | *Net1^loxp/+^* (n=4) |
| --- | --- | --- | --- |
| Ectopic OHCs per turn (Apex) | 1.60± 0.51 | 16.21 ± 3.25 | 1.50 ± 0.50 |
| Ectopic OHCs per turn (Middle) | 4.40 ± 2.58 | 15.71 ± 2.90 | 0.50 ± 0.50 |
| Ectopic OHCs per turn (Base) | 1.80 ± 1.80 | 5.21 ± 1.64 | 0.25 ± 0.25 |
| Ectopic IHCs per turn (Apex) | 4.20 ± 1.20 | 17.64 ± 2.64 | 1.25 ± 0.48 |
| Ectopic IHCs per turn (Middle) | 2.00 ± 0.63 | 16.14 ± 3.19 | 0.75 ± 0.48 |
| Ectopic IHCs per turn (Base) | 0.20 ± 0.20 | 8.50 ± 1.53 | 0.25 ± 0.25 |
| Total ectopic OHCs per cochlea | 7.80 ± 4.73 | 37.14 ± 6.58 | 2.25 ± 0.85 |
| Total ectopic IHCs per cochlea | 6.40 ± 0.87 | 42.29 ± 6.48 | 2.25 ± 1.03 |

Supplementary Table 4

Quantification of tdTomato^+^ HCs in P7 *Lgr5^CreER/+^Net1^loxp/loxp^tdTomato* mice and *Lgr5^CreER/+^tdTomato* mice. The n represents the number of mice used.

|  | *Lgr5^CreER/+^Net1^loxp/loxp^tdTomato* (n=4） | *Lgr5^CreER/+^tdTomato* (n=5) |
| --- | --- | --- |
| tdTomato^+^ OHCs per turn (Apex) | 386.25 ± 32.20 | 143 ± 12.83 |
| tdTomato^+^ OHCs per turn (Middle) | 55.25 ± 11.39 | 13.8 ± 3.95 |
| tdTomato+ OHCs per turn (Base) | 7.00 ± 2.71 | 7.00 ± 3.27 |
| tdTomato^+^ IHCs per turn (Apex) | 56.00 ±9.41 | 19.8 ± 2.72 |
| tdTomato^+^ IHCs per turn (Middle) | 0.25 ± 0.25 | 0.20 ± 0.22 |
| tdTomato^+^ IHCs per turn (Base) | 0.25 ± 0.25 | 0.20± 0.22 |
| tdTomato^+^ OHCs per cochlea | 448.5 ± 40.33 | 163.80 ± 13.84 |
| tdTomato^+^ IHCs per cochlea | 56.50 ± 9.15 | 20.20 ± 4.39 |
| tdTomato^+^ HCs per cochlea | 505.00 ± 47.32 | 184.00 ± 14.55 |
